# Reliability of ADDIS for diagnoses of substance use disorders according to ICD-10, DSM-IV and DSM-5: test-retest and inter-item consistency

**DOI:** 10.1186/s13011-015-0008-3

**Published:** 2015-04-02

**Authors:** Arne Gerdner, Lynn Wickström

**Affiliations:** Jönköping University, School of Health Sciences, P.O. Box 1026, SE 55111 Jönköping, Sweden

**Keywords:** Alcohol and drug dependence, Diagnoses, Assessment, Global reliability, Internal consistency

## Abstract

**Background:**

This study investigates test-retest and inter-item consistency of Alcohol Drog Diagnos InStrument (ADDIS), a structured interview to diagnose substance use disorders according to ICD-10, DSM-IV and DSM-5. ADDIS, the Swedish version of SUDDS, is the only instrument in Swedish that produces diagnostic proposals specific to all drug categories, and for all three diagnostic systems. Screening of stressful life events, anxiety, and depression is also included.

**Methods:**

Thirty patients at addiction treatment facilities were interviewed for diagnostic assessment and re-interviewed after one week.

**Results:**

ADDIS has excellent internal consistency. There is also very high test-retest correlation on number of fulfilled criteria for all diagnostic systems. Agreement of diagnostic proposals is substantial, mean absolute agreement is excellent, and mean systematic correlation is almost perfect.

**Conclusion:**

ADDIS is a reliable tool for specific diagnostic assessment of SUDs.

In Sweden, substance use disorders (SUDs) are diagnosed according to ICD-10 [1]. In research, and internationally, they may also be diagnosed according to DSM-IV [2] or the new DSM-5 [3]. As of today (2015), there are three instruments for diagnostic assessment of SUDs with manuals translated into Swedish: SCID-I (Structured Clinical Interview for the DSM-IV [axis I disorders]) [4], MINI (The Mini-International Neuropsychiatric Interview) [5,6] and ADDIS [7]. Of these three, ADDIS is the only one that provides detailed information on all substances as well as the only one that produces diagnostic proposals for all three diagnostic systems. With such ambitions, there should be high quality demands.

ADDIS (Alcohol Drog Diagnos InStrument) is the Swedish version of SUDDS (Substance Use Disorder Diagnostic Schedule) [8], a tool to diagnose SUDs. SUDDS was constructed as an improvement for NIMH-DIS (The National Institute of Mental Health Diagnostic Interview Schedule-Version II), a structured interview for the assessment of psychiatric disorders with high validity and reliability [9]. SUDDS is an event-oriented structured diagnostic interview that yields information for lifetime and current diagnosis (the past 12 months) of alcohol and other drug dependencies and abuse according to DSM. The latest version is SUDDS-5 [10].

ADDIS was translated and introduced to Sweden, with cultural adaptions, in 1987 by Wickström, who was also responsible for revising ADDIS making it compatible with DSM-III-R, DSM-IV and ICD-10. ADDIS consists of 75 questions, of which 47 are specific, behaviourally oriented questions on alcohol and other drugs based on DSM’s and ICD’s criteria for abuse/harmful use and dependence. Replies on specific questions are transferred to checklists for the diagnostic system used, i.e. ICD-10, DSM-IV or DSM-5, with columns for each drug category, resulting in specified current and lifetime diagnostic proposals. In addition, ADDIS includes smaller sections of screening for stressful experiences and problems concerning depression and anxiety.

Validity and reliability of SUDDS, studied in patients when assessed for inpatient alcohol and drug addiction treatment, was presented by Davis et al. [11]. SUDDS has good agreement with diagnostic assessment of experienced clinicians (Ƙ = .71 - .87). Test-retests show high correlation (R = .81 - .90), indicating high global consistence. When diagnoses based on SUDDS were compared to assessments by clinicians, the false negatives were less than one per cent and none were false positive [12]. Dependence and abuse appear as distinctive categories and reliability is similar in various ethnic groups (Afro-Americans, Hispanics, Native Americans and Caucasians), with internal consistency (α) for dependence varying between .93 and .97, and for abuse between .84 and .90 [13].

The Swedish ADDIS shows good construct validity concerning alcohol in two populations: a clinical population and a DWI population [14]. Principal Component Analysis found the two DSM-IV constructs – dependence and abuse – to be homogeneous with all items having factor loadings above .40 and acceptable explained variances. Separate analyses for the two populations and for women provided similar results. Discriminant validity was assessed by means of Discriminant Analysis. ADDIS could correctly classify 93.8 per cent of the two samples. Cronbach’s alpha is either satisfying or excellent in all analyses – both on criterion level and on item level, and for the different groups (clinical, DWI, women).

Sensitivity and specificity of ADDIS concerning alcohol and drug use disorders in comparison to the SCID and in comparison with a LEAD golden standard (Longitudinal, Expert, All Data) was studied by Gerdner et al. [15]. There is satisfactory agreement between ADDIS and SCID, although ADDIS is more sensitive than SCID. ADDIS demonstrates substantial to perfect agreement with the LEAD golden standard concerning alcohol as well as drugs, both lifetime and last year, and shows excellent to perfect overall sensitivity and specificity. Severity ratings (number of criteria) are almost perfectly in agreement with the LEAD standard.

Although internal consistency of ADDIS was presented concerning alcohol and DSM-IV, there is no previous study on the reliability of ADDIS concerning all drug categories and all three diagnostic systems. This present study is designed to measure the reliability of ADDIS, both as global consistency and as internal consistency, concerning the various drug categories. Internal consistency, or *inter-item consistency*, concerns how well various items of a test correlate with each other, while global consistency concerns how well the total test agrees with itself, if used at different occasions under same or similar circumstances. Global consistency can be studied either as *inter-rater reliability*, i.e. how well different raters agree with each other using the same instrument, or as *test-retest reliability*, i.e. the ability of a test to produce the same measure at different occasions under similar conditions. Test-retest is therefore the repeatability of a test, and is explored in repeating the test on the same individuals. Test-retest is sometimes found to be more critical, showing lower agreement, than inter-rater reliability in psychiatric assessment [16,17].

Test-retest reliability is desired for constructs that are not expected to change within a short time. Behaviours, e.g. drinking or using drugs, may differ from one day to another. An alcohol dependent person may one day be “on the wagon” and another day, after a relapse, be involved in binge drinking. Diagnoses, however, are supposed to be relatively stable. Instruments used for diagnostic assessment should therefore produce the same diagnostic proposal for the same person when assessed on a new occasion shortly after. These diagnoses should be repeatable within a time-span suitable for the condition assessed. Persons may recover, so that they no longer meet the criteria. SUDs diagnoses according to ICD-10 and DSM-IV are assessed as current diagnosis of dependence, substance abuse or harmful use, and in DSM-5 as substance use disorder if the person has met the criteria during the past 12 months. Test-retest should have a time-span between tests long enough for the test person not to remember previous replies when retested, but not so long that real change is likely to have occurred.

The aim here is to explore the reliability of ADDIS, as test-retest as well as inter-item consistency for the criteria and diagnostic proposals on SUDs according to ICD-10, DSM-IV and DSM-5, specific to various drug categories, and for its three screening tests concerning stress, anxiety and depression.

## Methods

The study was done as a quality assurance study in cooperation with alcohol and drug treatment facilities, where patients agreed to participate in a test-retest investigation of ADDIS. Trained interviewers among the treatment staff carried out the interviews. In this study, one week is regarded as a time-span during which recovery would not affect diagnoses and long enough for most persons not to remember previous replies. The first and second interviews were separated by approximately a week (mean 7.6 days, s.d. = 1.89). The two interview protocols, attached to each other, were sent to the researchers (i.e. the authors) without names or personal ID:s of the interviewed patients. Thus, patients were totally anonymous to the researchers, and only known to the treatment staff. This procedure was reviewed in the Research Ethics Committee of Mid Sweden University without objections.

Thirty patients (27 men and 3 women, mean age 37 ys [s.d. = 12.0]) agreed to participate. Nineteen were patients in three municipal residential treatment settings, 8 were in ambulatory community care organized by a probation office, and 3 were patients in opioid substitution treatment at a psychiatric clinic. Participation was voluntary, and patients were assured that there would be no negative consequences from the ADDIS assessment for participation in their respective treatment programme.

The participants had 9–16 years of education (median = 12). Twelve were married, 4 divorced and 14 never married. All reported having misused alcohol. I addition, 15 had misused (i.e. taken other than prescribed by a physician) tranquillizers or sleeping pills, and 10 had misused analgesic. Sixteen had misused cannabis, 11 amphetamine, 8 cocaine, 8 inhalants, 7 hallucinogens and 6 heroin.

All were interviewed, for all substances used, in order to examine SUDs criteria met in the three diagnostic systems – ICD-10 (research version), DSM-IV and DSM-5. When tabulated, fulfilled drug criteria and diagnoses are reported according to drug categorisations stipulated in the three diagnostic systems. In all these, analgesics and heroin are collapsed to opioids, while tranquilizers and sleeping pills are categorised as “sedatives, hypnotics and anxiolytics”. Inhalants are categorised as “volatile solvents”, and amphetamines are categorised as “other stimulants”, besides cocaine, while in DSM-5, amphetamines and cocaine are collapsed into “stimulants”.

In the statistical analyses two principal methods of test agreement are Cohen’s Kappa (К) and Gamma (γ). *К* estimates agreement when corrected for random agreement, varying from 1 in case of perfect agreement, to 0 which is not more than random agreement. Negative values exist when agreement is less than random. Kappa can be interpreted as follows: <.00 = “poor”, 0.00 - 0.20 = “slight”, 0.21 - 0.40 = “fair”, 0.41 - 0.60 = “moderate”, while 0.61-0.80 is “substantial” and >0.81 is “almost perfect” [18]. Gamma is a test of systematic correlation and is here interpreted in the same way as К. Absolute agreement is the percentage of protocols having identical outcome on the particular criterion or diagnostic proposal and is interpreted as follows: < 70% = “poor”, 70–79% “moderate”, 80–89% = “satisfactory”, 90–99% = “excellent”, 100% = “perfect”. Internal consistency is tested with Cronbach’s alpha (α), which is interpreted as “acceptable” if α ≥ .50, as “satisfactory” when α ≥ .70, and “excellent” when α ≥ .90 [19].

## Results

Although analyses were conducted for the assessment of both lifetime and current diagnoses with similar results, only the current will be presented here in order to save space.

### Internal consistency on item and criteria level

The basis of ADDIS diagnostic assessment on SUDs are 47 items, of which two were 6-point Likert-scale questions on quantity and frequency of alcohol use (but not other drugs) and 45 are questions on symptoms asked for all substance categories which can be answered as follows: 0 = Not used, 1 = No, 2 = Yes, lifetime, 3 = Yes, last year. Internal consistency using all relevant items is extremely high for each of these categories (alcohol: α = .95, all other drugs: α > .98). Since items are not used to create scales, but organized according to criteria of the diagnostic systems, ICD-10, DSM-IV and DSM-5, the internal consistency on criteria level is more relevant, and shown in Table 1 for the drug categories of each diagnostic system, respectively. The criteria of the diagnostic categories harmful use in ICD-10, and substance abuse in DSM-IV are added to the criteria of substance dependence in the table.

**Table 1:** **Internal consistency (α) on criteria level of substance use disorders for the drug categories of ICD-10, DSM-IV and DSM-5, respectively (n =30)**

	**ICD-10 (7 criteria) a)**	**DSM-IV (11 criteria) b)**	**DSM-5 (11 criteria)**	**Mean**
Alcohol	.87	.84	.87	.86
Sedatives, hypnotics, anxiolytics	.97	.95	.96	.96
Cannabis/cannabinoids	.97	.94	.95	.95
Cocaine c)	.94	.93	.94	.93
Amphetamines/other stimulants c)	.91	.93		
Opioids	.94	.95	.94	.94
Hallucinogens	.98	.90	.90	.93
Volatile solvents	.98	.89	.89	.92
Mean, all substances	.95	.92	.92	.93

a) Includes 6 criteria on dependence and 1 on harmful use. b) Includes 7 criteria on dependence and 4 on abuse. c) Cocaine and amphetamines/other stimulants are collapsed to *stimulants* in DSM-5.

All analyses show satisfactory or excellent internal consistency. For instance, the mean internal consistency across diagnostic systems range from an alpha of .86 for alcohol (lowest) to .96 for sedatives, hypnotics and anxiolytics (highest). The overall mean alpha was .93. Thus, there is strong evidence of inter-item reliability for all types of drugs in all three diagnostic systems.

### Test-retest correlations of number of fulfilled criteria

Systematic test-retest correlations on number of criteria met for various drugs according to the diagnostic systems are presented in Table 2. For ICD-10, agreement concerning the dichotomous variable harmful use was investigated with absolute as well as systematic agreement.

**Table 2:** **Systematic correlation (γ) between two assessments – test, and retest after one week – as to number of fulfilled criteria of substance use disorders according to the ICD-10, DSM-IV and DSM-5, respectively**

	**ICD-10**			**DSM-IV**		**DSM-5**
	**Substance dependence, 6 criteria**	**Harmful use, one dichotomous criterion**		**Substance dependence, 7 criteria**	**Substance abuse, 4 criteria**	**Substance use disorder, 11 criteria**
	**γ**	**%**	***К***	**γ**	**γ**	**γ**
Alcohol	.82	97	.84	.84	.80	.78
Sedatives, hypnotics, anxiolytics	.91	97	.93	.84	.93	.87
Cannabinoids a)	.97	97	.93	.96	.98	.94
Cocaine b)	.99	93	.79	.97	1.00	-
Other stimulants c)	.91	93	.83	.90	.93	.93
Opioids d)	.76	97	.92	.97	.95	.94
Hallucinogens	1.00	97	.65	.95	1.00	.88
Volatile solvents	1.00	93	.46	1.00	.93	.99
Mean, all substances	.92	96	.79	.93	.94	.90

a) Cannabis in DSM-IV; b) Cocaine, amphetamines and other stimulants are collapsed to stimulants in DSM-5; c) In ICD-10 this includes amphetamine and amphetamine-like substances (not caffeine), while in DSM-5, stimulants also include cocaine; d) Includes opiates as well as synthetic opioids, e.g. analgesics.

In addition, agreement concerning harmful use is tested with absolute agreement (%) as well as systematic agreement (К), (n = 30).

Systematic agreement of harmful use in ICD-10 range from moderate to almost perfect (К:s = .46 - .93), with the mean being borderline to almost perfect (.79). Systematic correlations of dependence criteria in ICD-10 range from substantial to perfect (γ:s = .76 - 1.00), with the mean being almost perfect (.92). Systematic correlation concerning number of dependence criteria met in DSM-IV were almost perfect or perfect (γ:s = .84 - 1.00), and for substance abuse criteria, they range from approaching almost perfect to being perfect (γ:s = .80 - 1.00). Systematic correlation on number of fulfilled DSM-5 criteria of substance use disorder range from substantial to almost perfect (γ:s = .78 - .99). Subsequently, systematic correlation on number of fulfilled criteria are very high for all three diagnostic systems. The mean systematic correlation range from .90 to .94 with a summary mean of .92.

### Test-retest agreement on diagnoses of SUDs

Based on number of various criteria met, test-retest reliability of diagnostic proposals according to ICD-10 is presented in Table 3 for each drug category.

**Table 3:** **Agreement between test and retest for ICD-10 diagnoses on substance use disorders (n = 30)**

	**Retest\test**	**No diagnose**	**Harmful use**	**Dependence**	**Agreement measures**
Alcohol	No diagnose	3	0	1	К = .72 (p < 0.001)
	Harmful use	0	0	1	γ = 1.00 (p = 0.041)
	Dependence	0	0	25	Absolute agreement = .93
Sedatives, hypnotics, anxiolytics	No diagnose	16	0	0	К = .93 (p < 0.001)
	Harmful use	0	0	0	γ = 1.00 (p < 0.001)
	Dependence	1	0	13	Absolute agreement = .97
Cannabinoids	No diagnose	18	0	0	К = .87 (p < 0.001)
	Harmful use	1	0	0	γ = 1.00 (p < 0.001)
	Dependence	0	1	10	Absolute agreement = .93
Cocaine	No diagnose	23	1	0	К = .68 (p < 0.001)
	Harmful use	1	1	0	γ = .94 (p = 0.019)
	Dependence	1	0	3	Absolute agreement = .90
Other stimulants	No diagnose	21	1	0	К = .50 (p < 0.001)
	Harmful use	1	0	2	γ = .92 (p < 0.001)
	Dependence	1	1	3	Absolute agreement = .80
Opioids	No diagnose	21	1	0	К = .77 (p < 0.001)
	Harmful use	0	1	2	γ = 1.00 (p < 0.001)
	Dependence	0	0	5	Absolute agreement = .90
Hallucinogens	No diagnose	28	0	0	К = .31 (p = 0.021)
	Harmful use	1	0	0	γ = 1.00 (n.s.)
	Dependence	0	1	0	Absolute agreement = .93
Volatile solvents	No diagnose	27	1	0	К = .47 (p = 0.001)
	Harmful use	1	0	0	γ = .93 (n.s.)
	Dependence	0	0	1	Absolute agreement = .93

Systematic agreement varied from fair (hallucinogens) and moderate (volatile solvents, other stimulants) to substantial (alcohol, cocaine and opioids) and almost perfect (sedatives/hypnotics/anxiolytics and cannabinoids). Mean systematic agreement was substantial (Ƙ = .66). Absolute agreements were all satisfactory or excellent (range: .80 - .97; mean .91), and all systematic correlations were almost perfect or indeed perfect (γ range: .92 - 1.00, mean .97). Table 4 presents test-retest agreement concerning DSM-IV diagnoses.

**Table 4:** **Agreement between test and retest for DSM-IV diagnoses on substance use disorders (n = 30)**

	**Retest\test**	**No diagnose**	**Abuse**	**Dependence**	**Agreement measures**
Alcohol	No diagnose	2	0	2	К = .44 (p = 0.002)
	Abuse	0	0	1	γ = .89 (n.s.)
	Dependence	0	1	24	Absolute agreement = .87
Sedatives, hypnotics, anxiolytics	No diagnose	16	0	0	К = .93 (p < 0.001)
	Abuse	0	0	0	γ = 1.00 (p < 0.001)
	Dependence	1	0	13	Absolute agreement = .97
Cannabis	No diagnose	17	0	1	К = .79 (p < 0.001)
	Abuse	1	0	0	γ = .98 (p < 0.001)
	Dependence	1	0	10	Absolute agreement = .90
Cocaine	No diagnose	24	1	0	К = .89 (p < 0.001)
	Abuse	0	1	0	γ = 1.00 (p = 0.003)
	Dependence	0	0	4	Absolute agreement = .97
Amphetamine (or	No diagnose	23	0	0	К = .82 (p < 0.001)
amphetamine-like)	Abuse	0	0	1	γ = .99 (p < 0.001)
	Dependence	0	1	5	Absolute agreement = .93
Opioids	No diagnose	20	1	2	К = .77 (p < 0.001)
	Abuse	0	1	0	γ = .97 (p < 0.001)
	Dependence	0	0	6	Absolute agreement = .90
Hallucinogens	No diagnose	28	0	0	К = .31 (p = 0.021)
	Abuse	1	0	0	γ = 1.00 (n.s.)
	Dependence	0	1	0	Absolute agreement = .93
Volatile solvents	No diagnose	27	1	0	К = .47 (p = 0.001)
	Abuse	1	0	0	γ = .93 (n.s.)
	Dependence	0	0	1	Absolute agreement = .93

Here too, systematic agreement varied from fair to almost perfect. Differences, compared to ICD-10, were lower agreement on alcohol (moderate) and cannabis (substantial), while agreement on cocaine and amphetamines increased (almost perfect). Mean systematic agreement approached substantial (Ƙ = .69). Absolute agreements were all satisfactory or excellent (range: .87 - .97; mean .93), and all systematic correlations were almost perfect or indeed perfect (γ range: .89 - 1.00, mean .97). Table 5 presents the agreement concerning DSM-5 diagnoses.

**Table 5:** **Agreement between test and retest for DSM-5 diagnoses on substance use disorders (n = 30)**

	**Retest\test**	**No diagnose**	**Mild**	**Moderate**	**Severe**	**Agreement measures**
Alcohol	No diagnose	2	0	0	2	К = .71 (p < 0.001)
	Mild	0	0	1	0	γ = .88 (p = 0.001)
	Moderate	0	0	4	1	Absolute
	Severe	0	0	0	20	agreement = .87
Sedatives, hypnotics, anxiolytics	No diagnose	16	0	0	0	К = .81 (p < 0.001)
	Mild	0	0	0	0	γ = .98 (p < 0.001)
	Moderate	0	0	0	1	Absolute
	Severe	1	0	1	11	agreement = .90
Cannabis	No diagnose	15	1	0	0	К = .83 (p < 0.001)
	Mild	1	2	0	0	γ = .97 (p < 0.001)
	Moderate	1	0	0	0	Absolute
	Severe	0	0	0	10	agreement = .90
Stimulants	No diagnose	22	0	0	0	К = .67 (p < 0.001)
	Mild	1	0	0	1	γ = .98 (p < 0.001)
	Moderate	0	0	0	0	Absolute
	Severe	0	0	2	4	agreement = .87
Opioids	No diagnose	21	1	0	0	К = .85 (p < 0.001)
	Mild	0	2	0	0	γ = 1.00 (p < 0.001)
	Moderate	0	0	0	1	Absolute
	Severe	0	0	0	5	agreement = .93
Hallucinogens	No diagnose	27	1	0	0	К = .21 (n.s.)
	Mild	1	0	0	0	γ = .93 (n.s.)
	Moderate	0	0	0	0	Absolute
	Severe	0	1	0	0	agreement = .90
Volatile solvents	No diagnose	27	1	0	0	К = .47 (p = 0.001)
	Mild	1	0	0	0	γ = .93 (n.s.)
	Moderate	0	0	0	0	Absolute
	Severe	0	0	0	1	agreement = .93

As for the other two diagnostic systems, systematic agreement varied from fair to almost perfect. In comparison to DSM-IV, alcohol improved from moderate to substantial; cannabis was – as in ICD-10 – almost perfect; and stimulants (cocaine as well as amphetamines/other stimulants) were substantially in agreement. Opioids improved to almost perfect. For sedatives/hypnotics/anxiolytics, hallucinogens and volatile solvents findings were stable across diagnostic systems. As with ICD-10 and DSM-IV, mean systematic agreement of DSM-5 diagnoses was substantial (Ƙ = .65). Absolute agreement ranged from substantial to excellent (87 to 93%, mean = 90), and all systematic correlations were almost perfect or perfect (γ range: .88 - 1.00, mean .95).

### Summing up drug categories

Findings concerning inter-item consistency, and test-retest consistency on criteria as well as diagnostic proposals according to ICD-10, DSM-IV and DSM-5 are summed up for each drug category.

#### Alcohol

Internal consistency is satisfactory (α = .84 to .87). Systematic correlation between test and retest *on number of fulfilled criteria* range from substantial to almost perfect (γ = .78 to .84). Absolute agreement *on diagnostic level* is satisfactory to excellent (87 to 93%), while systematic agreement range from moderate to substantial (К = .44 to .72), and systematic correlation is almost perfect or indeed perfect (γ = .88 to 1.00).

#### Sedatives, hypnotics, anxiolytics

Internal consistency is excellent (α = .95 to .97). Systematic correlation on criteria level between test and retest is almost perfect (γ = .84 to .93). On diagnostic level absolute agreement between test and retest is satisfactory to excellent (90 to 97%), while systematic agreement is almost perfect (К = .81 to .93), and systematic correlation is almost perfect or perfect (γ = .98 to 1.00).

#### Cannabis/cannabinoids

Internal consistency is excellent (α = .94 to .97). Systematic correlation on criteria level between test and retest is almost perfect (γ = .94 to .98). On diagnostic level absolute agreement between test and retest is excellent (90 to 93%), while systematic agreement is substantial or almost perfect (К = .79 to .87), and systematic correlation is almost perfect or perfect (γ = .97 to 1.00).

#### Cocaine, amphetamine and other stimulants

Internal consistency is excellent (α = .91 to .94). Systematic correlation on criteria level between test and retest is almost perfect or perfect (γ = .90 to 1.00). On diagnostic level absolute agreement between test and retest is satisfactory to excellent (80 to 97%), while systematic agreement range from moderate to almost perfect (К = .50 to .89), and systematic correlation is almost perfect or perfect (γ = .92 to 1.00).

#### Opioids

Internal consistency is excellent (α = .94 to .95). Systematic correlation on criteria level between test and retest is substantial to almost perfect (γ = .76 to .97). On diagnostic level absolute agreement between test and retest is excellent (90 to 93%), while systematic agreement is substantial or almost perfect (К = .77 to .85), and systematic correlation is almost perfect or perfect (γ = .97 to 1.00).

#### Hallucinogens

Internal consistency is excellent (α = .90 to .98). Systematic correlation on criteria level between test and retest is almost perfect or indeed perfect (γ = .88 to 1.00). On diagnostic level absolute agreement between test and retest is excellent (90 to 93%), while systematic agreement is only fair (К = .21 to .31), and the systematic correlation is almost perfect or perfect (γ = .93 to 1.00).

#### Volatile solvents

Internal consistency is satisfactory to excellent (α = .89 to .98). Systematic correlation on criteria level between test and retest is almost perfect to perfect (γ = .93 to 1.00). On diagnostic level absolute agreement between test and retest is excellent (all 93%), while systematic agreement is moderate (all К = .47), and systematic correlation is almost perfect (all γ = .93).

### Reliability of scales concerning stress, anxiety and depression

The scales for screening stressful life events, anxiety and depression were analysed for inter-item as well as for test-retest consistency (n = 27). Internal consistency was satisfactory for stress (α = .72), and excellent for anxiety and depression (α = .90 and .96, respectively). Test-retest showed almost perfect systematic correlation for all scales (stressful life events: γ = .85; anxiety: γ = .96, and depression: γ = .92).

## Discussion

The study has some obvious limitations. It was conducted without external funding. A convenience sample was used. The cooperating treatment facilities conducted interviews on their own budgets. The sample is therefore smaller than would have been preferred, but about the same as in similar studies e.g. [20-22].

Due to the variety of patients enrolled, and despite the relatively small sample, it was possible to include all types of drugs that might be assessed using ADDIS with more than five cases for each condition, a cut-off used in e.g. Zanarini et al. [16]. These drugs were, as expected, used by different numbers of patients resulting in some skewness in number of cases vs. non-cases. Systematic agreement (Cohen’s Kappa) and systematic correlation (Gamma) handles such skewness to some degree. While Kappa tends to be lower than Gamma on skewed categories, Gamma tends to be more conservative in testing statistical significance (p-values). We therefore present both of these.

The relatively lower Kappa values for hallucinogens, volatile solvents and alcohol may be related to skewness of these variables – with relatively few who used hallucinogens and volatile solvents and with almost everyone having some problem concerning alcohol. The problem was not however indicated by absolute agreement or systematic correlation.

Taking all drug categories together, ADDIS has an excellent overall mean internal consistency of .93. Test-retest correlation on number of fulfilled criteria is very high for all three diagnostic systems. Their mean systematic correlations on criteria level for various drugs and diagnostic systems are almost perfect (.92 - .94). At the level of diagnostic proposals across all three diagnostic systems, mean systematic agreement is substantial (Ƙ = .65 - .69), while mean absolute agreement is excellent (90 - 93%), and mean systematic correlation is almost perfect (γ = .95-.97).

Both internal and global consistencies are equivalent to or better than previously found concerning: SUDDS for DSM-III-R and DSM-IV [11,13]; different versions of SCID for DSM-IV [16,20,23-25] different versions of MINI for DSM-III-R and DSM-IV [17,22]; and the Composite International Diagnostic Interview (CIDI) for ICD-10 and DSM-III-R [26].

In addition, screening tests on stressful life events, anxiety and depression showed satisfactory to excellent internal consistency (α = .72, .90 and .96 respectively), and almost perfect global consistency for all scales (γ = .84 - .96).

Despite the small sample, the reliability findings are sufficiently striking so as to indicate that the ADDIS consistently provides substance specific diagnostic documentation. ADDIS is the only currently used instrument in Swedish, which is capable of providing substance specific diagnostic information in a relatively brief interview to be practical in routine clinical practice. Further research with a larger and more diverse sample is indicated to extend the findings of the current study.

## References

[1] WHO (1993). The ICD-10 Classification of Mental and Behavioural Disorders. Diagnostic criteria for research.

[2] APA (1994). Diagnostic and Statistical Manual of Mental Disorders, 4th ed. rev. (DSM-IV).

[3] APA (2013). The Diagnostic and statistical manual of mental disorders – fifth edition. (DSM-5).

[4] First MB, Gibbon M, Spitzer RL, Williams J, Benjamin LS (1999). Handbok—SCID-I och SCID-II för DSM-IV. Svensk bearbetning av Jörgen Herlofsson.

[5] Sheehan D, Janavs J, Harnett-Sheehan K, Sheehan M, Gray C, Lecrubier Y (2010). MINI Internationella Neuropsykiatriska Intervju (M.I.N.I. Swedish Translation Version 6.0.0, DSM-IV).

[6] Sheehan DV, Lecrubier Y, Sheehan KH, Janavas J, Weiller E, Keskiner A (1998). The Mini-International Neuropsychiatric Interview (M.I.N.I.): The development and validation of a structured diagnostic psychiatric interview for DSM-IV and ICD-10. J Clin Psychiatry.

[7] ADDIS (Alkohol Drog Diagnos InStrument (2011). Manual, version 09/2011. Åre: Dahl & Dahl AB.

[8] Harrison PA, Hoffmann NG (1985). SUDDS: Substance Use Disorder Diagnostic Schedule.

[9] Harrison PA, Hoffmann NG (1989). SUDDS - Substance Use Disorder Diagnostic Schedule Manual.

[10] Hoffmann NG, Harrison PA. SUDDS-5 Manual – Substance Use Disorder Diagnostic Schedule - 5. Carson City, NV: The Change Companies; 2013.

[11] Davis LJ, Hoffmann NG, Morse RM, Luehr J (1992). Substance use disorder diagnostic schedule and an interviewer-administered format. Alcoholism: Clin Exp Res.

[12] Hoffmann NG, Harrison PA (1996). SUDDS-IV (Substance use disorder diagnostic Schedule-IV) Manual.

[13] Hoffmann NG, Hoffmann TD (2003). Construct validity for alcohol dependence as indicated by the SUDDS-IV. Subst Use Misuse.

[14] Gerdner A (2009). Diagnosinstrument för beroende och missbruk—Granskning av ADDIS validitet och interna konsistens gällande alkoholproblem. Nordisk Alkohol- Narkotikatidskrift.

[15] Gerdner A, Kestenberg J, Edvinsson M (2015). Validity of the Swedish SCID and ADDIS diagnostic interviews for substance use disorders: Sensitivity and specificity compared with a LEAD golden standard. Nord J Psychiatry.

[16] Zanarini MC, Skodol AE, Bender D, Dolan R, Sanislow C, Schaefer E (2000). The Collaborative Longitudinal Personality Disorders Study: Reliability of Axis I and II diagnoses. J Pers Disord.

[17] Otsubo T, Tanaka K, Koda R, Shinoda J, Sano N, Tanaka S (2005). Reliability and validity of Japanese version of the Mini-International Neuropsychiatric Interview. Psychiatry Clin Neurosci.

[18] Landis JR, Koch GG (1977). The measurement of observer agreement for categorical data. Biometrics.

[19] Streiner DL, Normann GR (1989). Health measurement scales: a practical guide to their development and use.

[20] Schneider B, Maurer K, Sargk D, Heiskel H, Weber B, Frölich L (2004). Concordance of DSM-IV Axis I and II diagnoses by personal and informant’s interview. Psychiatry Res.

[21] Mordal J, Gundersen Ø, Bramnes JG (2010). Norwegian version of the Mini-International Neuropsychiatric Interview: Feasibility, acceptability and test-retest reliability in an acute psychiatric ward. Eur Psychiatry.

[22] Kadri N, Agoub M, Gnaoui S, Mchichi Alami K, Hergueta T, Moussaoui D (2005). Moroccan colloquial Arabic version of the Mini International Neuropsychiatric Interview (MINI): qualitative and quantitative validation. Eur Psychiatry.

[23] Smith DC, Huber DL, Hall JA (2005). Psychometric Evaluation of the Structured Clinical Interview for DSM-IV Childhood Diagnoses (KID-SCID). J Hum Behav Soc Environ.

[24] Lobbestael J, Leurgans M, Arntz A (2011). Inter-rater reliability of the Structured Clinical Interview for DSM-IV Axis I Disorders (SCID I) and Axis II Disorders (SCID II). Clin Psychol Psychother.

[25] Williams JBW, Gibbon M, First MB, Spitzer RL, Davies M, Borus J (1992). The Structured Clinical Interview for DSM-III-R (SCID): II. Multisite Test-Retest Reliability Arch Gen Psychiatry.

[26] Rubio-Stipec M, Peters L, Andres G (1999). Test-retest reliability of the computerized CIDI (CIDI-auto): Substance abuse modules. Subst Abus.

